# CLAIMS-BASED FRAILTY INDEX (CFI) AS A MEASURE OF DEMENTIA SEVERITY IN MEDICARE BENEFICIARIES WITH ADRD

**DOI:** 10.1093/geroni/igac059.2820

**Published:** 2022-12-20

**Authors:** Chan Mi Park, Stephanie Denise Sison, Ellen McCarthy, Natalia Gouskova, Dae Kim

**Affiliations:** Hebrew SeniorLife, Roslindale, Massachusetts, United States; Beth Israel Deaconess Medical Center, Brookline, Massachusetts, United States; Hebrew SeniorLife, Roslindale, Massachusetts, United States; Hebrew SeniorLife, Roslindale, Massachusetts, United States; Hebrew SeniorLife, Roslindale, Massachusetts, United States

## Abstract

Lack of a dementia severity measure in administrative claims data is a major barrier in Alzheimer’s Disease and Related Dementias (ADRD) research using such data sources. A claims-based frailty index (CFI; range 0 to 1, higher scores indicating greater frailty) is a validated measure of frailty that can be calculated from administrative claims data and is correlated with functional status. We conducted a cross-sectional study to examine whether CFI can be a claims-based measure to differentiate dementia severity in 814 participants with ADRD with linked to Medicare claims in the National Health and Aging Trends Study (NHATS). We estimated the Functional Assessment Staging Test (FAST) scale (3: mild cognitive impairment; 4: mild dementia; 5: moderate dementia; 6–7 moderate-to-severe dementia) using NHATS variables and calculated CFI from Medicare claims in the prior 12 months. The prevalence of FAST stage 5 or higher was 244 (weighted percentage, 25.9%). The C-statistic of CFI to identify FAST stage 5 or higher was 0.78 [95% CI:0.73–0.82], with a CFI cut-point of 0.3 achieving the maximum sensitivity 72.5% and specificity 67.5%. The equipercentile linking procedure showed that CFI scores of 0.30, 0.35, and 0.40 corresponded to FAST stages 4, 5, and 6, respectively. Our results support the utility of a CFI as a proxy of dementia severity in administrative claims data.

